# Renal impairment is associated with increased risk of mortality in patients with cirrhosis: A retrospective cohort study

**DOI:** 10.1097/MD.0000000000014475

**Published:** 2019-02-08

**Authors:** Takeji Umemura, Satoru Joshita, Soichiro Shibata, Ayumi Sugiura, Tomoo Yamazaki, Naoyuki Fujimori, Akihiro Matsumoto, Eiji Tanaka

**Affiliations:** a Department of Medicine, Division of Hepatology and Gastroenterology, Shinshu University School of Medicine; b Research Center for Next Generation Medicine, Shinshu University, Matsumoto, Nagano, Japan.

**Keywords:** cirrhosis, creatinine, prognosis, renal impairment, survival

## Abstract

Although renal impairment is a frequent complication in cirrhosis that is associated with a poor prognosis, little has been reported on the clinical significance of renal impairment in cirrhosis in Japan. This retrospective study assessed the impact of renal impairment on mortality in Japanese cirrhosis patients taking conventional diuretics.

A total of 157 patients with cirrhosis receiving diuretic treatment were evaluated for the presence and status of renal impairment, defined as an increase in serum creatinine of ≥ 0.3 mg/dL or by ≥ 50%, and then classified according to the International Club of Ascites (ICA)-Acute Kidney Injury (AKI) staging system.

Eighty of 157 (51%) patients fulfilled the criteria for renal impairment. Thirty-four (43%) patients had ICA-AKI stage 1, 32 (40%) stage 2, and 14 (18%) stage 3. Multivariate analysis revealed female gender (hazard ratio [HR] = 0.407, 95% confidence interval = 0.193–0.857; *P* = .018), ALT ≥35 IU/L (HR = 3.841, 95% confidence interval = 1.785–8.065; *P* = .001), and the presence of renal impairment (HR = 4.275, 95% confidence interval = 1.962–9.312; *P* < .001) as independent factors significantly increasing the risk of mortality. Cumulative survival rates increased significantly with ICA–AKI stage (log-rank test, *P* = .009).

Renal impairment was a predictive marker of mortality in Japanese patients with cirrhosis. Stratification according to ICA–AKI criteria of kidney function impairment may be a good prognostic indicator of cirrhosis outcome.

## 1 Introduction

Cirrhosis is defined as the histological development of regenerative nodules surrounded by fibrous bands in response to chronic liver injury that leads to portal hypertension, end-stage liver disease, and hepatocellular carcinoma (HCC).^[1,2]^ Occurring in 20% of hospitalizations,^[3]^ acute kidney injury (AKI) is a frequent complication in cirrhosis patients associated with significant mortality^[4–7]^; one meta-analysis reported a 6.38-fold increased risk of death in patients with AKI.^[8]^

Although several diagnostic systems have been established for renal impairment classification in cirrhosis,^[8–10]^ it is difficult to precisely quantify the clinical impact of renal impairment. The International Club of Ascites (ICA) developed new criteria for AKI, defining it as an abrupt reduction in renal function in critically ill patients.^[11]^ The ICA classified renal impairment into three grades based on a rise in serum creatinine over 48 hours accompanied with mild (stage 1) to severe (stage 3) stages of AKI. Other reports have shown that progression of AKI to higher stages was independently associated with a poor prognosis in cirrhosis in Europe and the United States,^[12–15]^ although little is known on the clinical impact of AKI in cirrhosis patients in Japan. The objective of this study was to assess the prevalence of renal impairment in Japanese cirrhosis patients taking conventional diuretics and identify the impact of renal impairment status on mortality.

## 2 Methods

### 2.1 Subjects

A total of 157 consecutive patients with cirrhosis who had been receiving a diuretic regimen at Shinshu University Hospital between January 2003 and September 2013 were enrolled in this retrospective study. The racial background of all patients was Japanese. The protocol of this investigation was approved by the ethics committee of the Shinshu University School of Medicine in accordance with the Helsinki Declaration (No. 2712). All data were gathered in the context of standard practice from clinical patient records without the need for informed consent and were anonymized and stored in a protected database. The diagnosis of cirrhosis was based on histological examination (*n* = 25) and/or clinical, biochemical, and imaging findings (*n* = 132). No patients were diagnosed by transient elastography. Demographic, clinical, and laboratory data as well as score for Model for End-Stage Liver Disease (MELD) were obtained for each patient at the start of diuretic treatment. MELD score was calculated as reported previously.^[16]^ Exclusion criteria included prior kidney disease (baseline creatinine > 3.0 mg/dL), acute or chronic renal replacement therapy at the time of enrollment, estimated life expectancy of less than three days, and other known causes of renal insufficiency, such as glomerulonephritis or hydronephrosis.

Clinical data contained information about hepatic edema, gastro-esophageal varices, ascites, hepatic encephalopathy, and other complications of cirrhosis. The presence of ascites was determined by ultrasonography or computed tomography. Hepatitis B surface antigen, anti-hepatitis C virus (HCV) antibody, and HCV RNA (when positive for anti-HCV antibody) were evaluated to identify persistent hepatitis B virus (HBV) or HCV infections.^[17,18]^ Autoimmune hepatitis^[19]^ and primary biliary cholangitis (PBC)^[20]^ were diagnosed using histological examination and serological testing, as reported previously. Alcoholic liver disease (ALD) and non-alcoholic steatohepatitis (NASH) were determined using conventional methods.

### 2.2 Definition of renal impairment

Renal impairment was defined according to the ICA–AKI criteria. AKI was considered to exist for an increase in serum creatinine of ≥0.3 mg/dL or ≥50% over baseline during treatment with diuretics, which was defined as the most recent stable serum creatinine value available within the 3 months preceding therapy. AKI stages were defined as follows: stage 1 = increase in serum creatinine of ≥ 0.3 mg/dL or increase of 1.5-fold to 2-fold from baseline; stage 2 = increase in serum creatinine of 2-fold to 3-fold from baseline; stage 3 = increase in serum creatinine of >3-fold from baseline or serum creatinine of ≥ 4.0 mg/dL with an acute increase of ≥ 0.3 mg/dL or initiation of renal replacement therapy. The diagnostic criteria of hepatorenal syndrome type (HRS)-AKI were defined using ICA criteria as previously reported.^[11]^

### 2.3 Statistical analysis

The Mann–Whitney *U* test was used to analyze continuous variables. Fisher's exact and Pearson's chi-square tests were adopted for analysis of categorical data. To identify potential factors associated with death, univariate and multivariate analyses were conducted using the Cox proportional-hazards regression model. Comparisons of renal impairment status among patients were calculated using Kaplan–Meier statistics, and differences between groups were analyzed using the log-rank test. A *P* value of less than .05 was considered to be statistically significant. All statistical analyses were performed using IBM SPSS statistics software version 21.0.

## 3 Results

**Figure 1:**
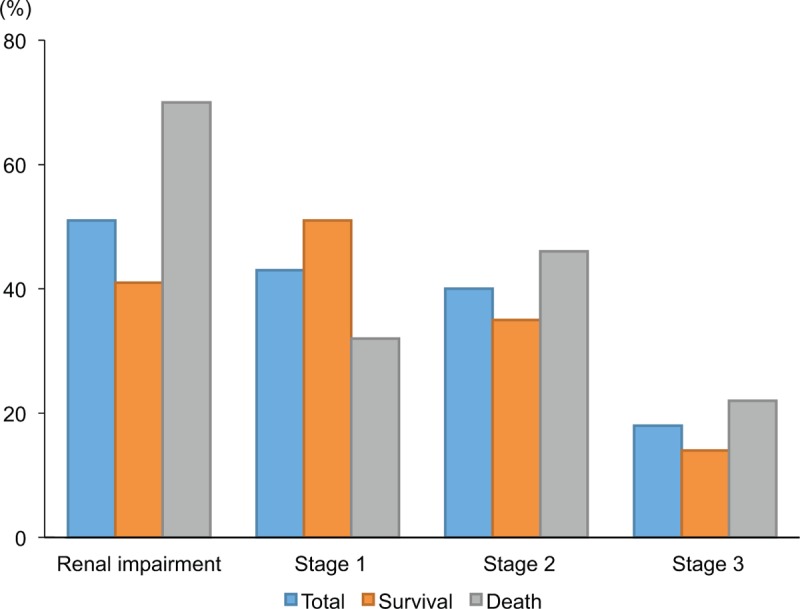
Prevalence of renal impairment in patients with cirrhosis.

The baseline demographic, clinical, and laboratory data of the patients included in this study are shown in Table 1. Median age was 60 years, and 45% of subjects were male. The median follow-up periods were 522 months. The etiology of cirrhosis was hepatitis C in 59% of patients, hepatitis B in 13%, NASH in 9%, ALD in 6%, PBC in 6%, and cryptogenic in 6%. Complications of leg edema were present in 69% of patients, gastro-esophageal varices in 61%, ascites in 51%, hepatic encephalopathy in 17%, and spontaneous bacterial peritonitis in 1%. With regard to diuretics, a combination of spironolactone and furosemide was given to 50% of patients, spironolactone alone to 27%, furosemide alone to 21%, and others to 2%. Interferon-based therapy and nucleo(s)tide analogs were taken by 25% of patients with HCV and 29% with HBV. Branched-chain amino acid granules were administered to 65% of all patients. Among the 157 patients enrolled, 80 (51%) fulfilled the ICA–AKI criteria (Fig. 1). Forty-three percent of patients had stage 1, 40% had stage 2, and 18% had stage 3. Among 44 patients with renal impairment and ascites, only 3 fulfilled the ICA diagnostic criteria for HRS-AKI due to albumin treatment restrictions prior to 2014.

**Table 1:**
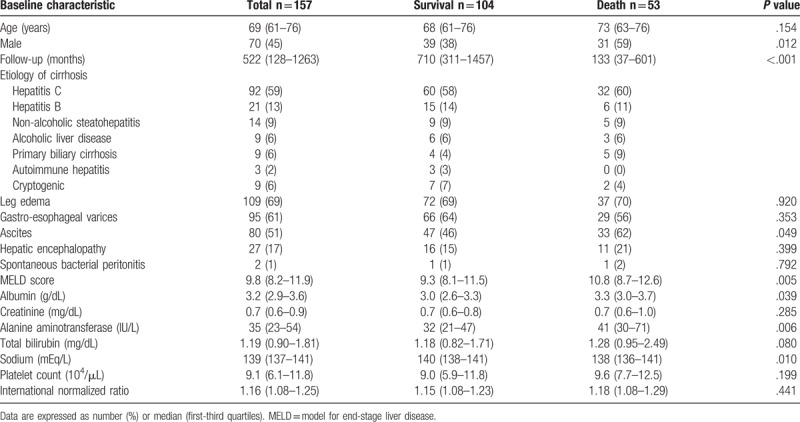
Baseline characteristics at enrollment and comparison of survival and death patients.

The patients were divided into two groups according to the absence or presence of renal impairment and their clinical features at the start of diuretics compared (Table 2). Univariate analysis revealed that patients with renal impairment had a higher peak creatinine (2.0 vs 0.8 mg/dL; *P* < .001), while no significant associations were detected for cirrhosis etiology or complications. The type of diuretic medication did not factor significantly between the renal impairment (+) and renal impairment (−) groups as well (spironolactone and furosemide: 48% vs 55%, respectively; spironolactone only: 21% vs 21%, respectively; furosemide only: 31% vs 24%, respectively).

**Table 2:**
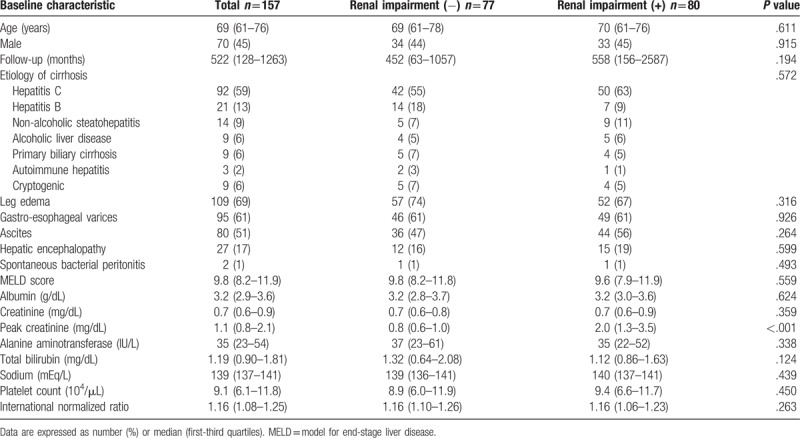
Comparison of characteristics between patients without and with renal impairment.

We next focused on the effect of renal impairment status on mortality. Of the 157 patients, 53 (34%) died from liver-related causes (30 from HCC, 17 from liver failure, and 6 from bleeding esophageal varices) during the follow-up period. No patients received liver transplantation. As shown in Table 3, non-surviving patients displayed a male preponderance (59% vs 38%; *P* = .012), higher incidence of ascites (62% vs 46%; *P* = .049), higher MELD score (10.8 vs 9.3; *P* = .005), higher alanine aminotransferase level (41 vs 32 IU/L; *P* = .006), lower albumin level (3.0 vs 3.3 mg/dL; *P* = .039), and lower sodium level (138 vs 140 mEq/L; *P* = .010). The prevalence of renal impairment in patients who died was significantly higher than that in survivors (70% vs 41%; *P* = .001) (Fig. 1). Higher AKI stage was significantly associated with mortality (*P* = .002). Multivariate Cox regression analysis of all factors identified as associated with survival in univariate analysis showed that female gender (hazard ratio [HR] = 0.407; 95% CI: 0.193–0.857; *P* = .018), ALT ≥ 35 IU/L (HR = 3.841; 95% CI: 1.785–8.265; *P* = .001), and the presence of renal impairment (HR = 4.275; 95% CI: 1.962–9.312; *P* < .001) were all significant independent variables associated with an increased risk of mortality (Table 3).

**Table 3:**
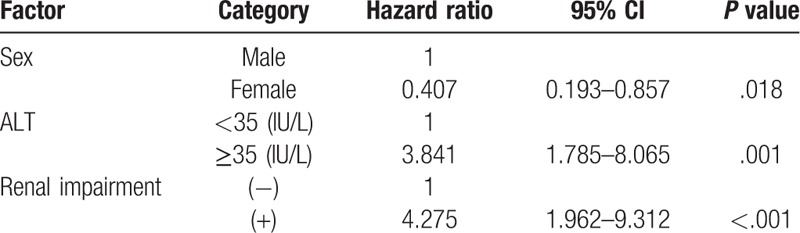
Multivariate analysis of predictive factors of mortality in cirrhosis.

**Figure 2:**
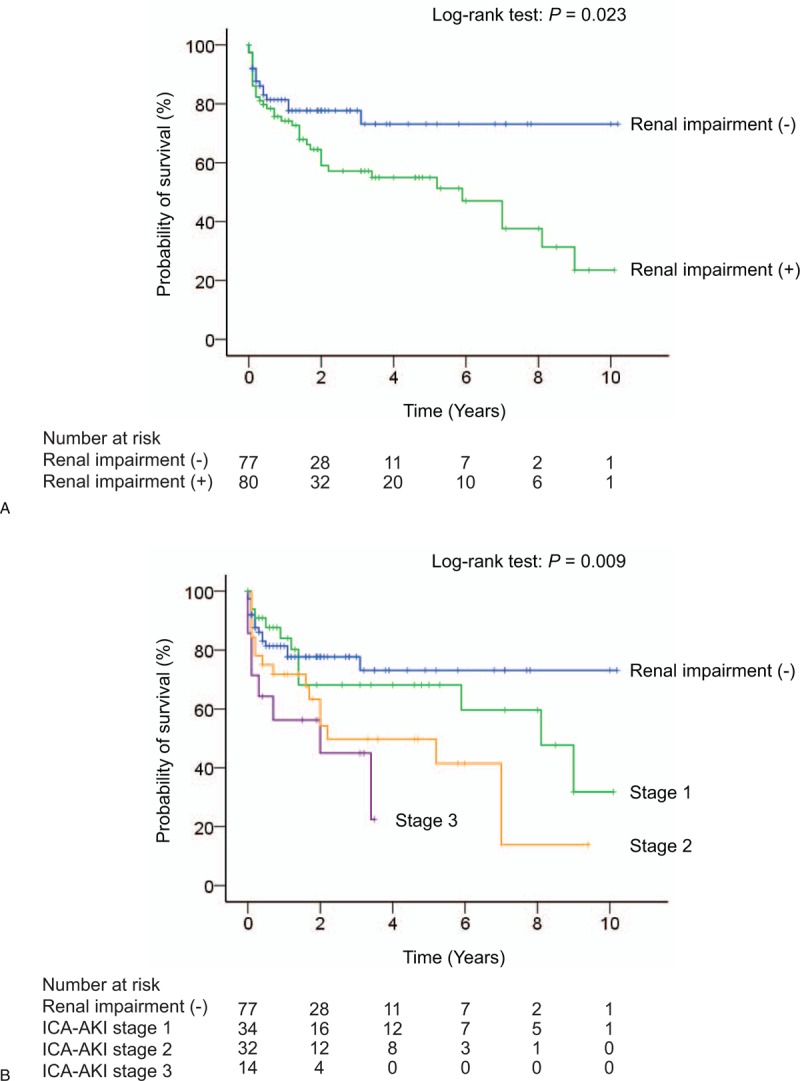
Kaplan–Meier overall survival curves based on renal impairment status. (A) Survival curves of patients classified according to the presence or absence of renal impairment. (B) Survival curves of patients classified according to the absence of renal impairment and ICA–AKI stage (no renal impairment vs stage 1, *P* = .493; no renal impairment vs stage 2, *P* = .012; no renal impairment vs stage 3, *P* = .007; stage 1 vs stage 2, *P* = .096; stage 1 vs stage 3, *P* = .030; stage 2 vs stage 3, *P* = .28).

Kaplan–Meier survival estimates of patients divided into renal impairment and non-renal impairment groups demonstrated a significant difference (log-rank test; *P* = .023) (Fig. 2A). Moreover, mortality increased with ICA–AKI stage (log-rank test; *P* = .009) (Fig. 2B), although there were no remarkable differences in survival between patients with ICA–AKI stages 1 and 2 (*P* = .096) or 2 and 3 (*P* = .285).

## 4 Discussion

The present study of Japanese cirrhosis patients receiving diuretics revealed that almost half of individuals developed renal impairment as assessed by the ICA–AKI criteria, which was similar to other reports.^[7,12]^ As the development of renal impairment in cirrhosis was also strongly associated with mortality, our findings support those from previous studies that emphasize the relevance of kidney function when determining prognosis in cirrhosis.^[6–8,12,15,21–23]^

We observed that mortality gradually increased with ICA–AKI stage and that the presence of renal impairment was an independent risk factor of mortality. There were significant differences in survival between patients without renal impairment and those with ICA–AKI stage 2 (*P* = .012) or 3 (*P* = .007). Moreover, survival in patients with ICA–AKI stage 1 was significantly higher than that in patients with stage 3 (*P* = .030). However, survival in patients with ICA–AKI stage 1 did not differ remarkably from that in patients with ICA–AKI stage 2 (*P* = .096). These results were not consistent with previous studies^[12,13]^ and may have been due to variations in patient selection, sample size differences, and ethnic variability.

Serum creatinine is an inexpensive and widely established marker of kidney function in clinical practice. However, creatinine is considered as a late marker of decreased renal function since increases in serum values are delayed compared with renal injury, meaning that significant renal disease can exist with minimal or no alterations in serum creatinine because of enhanced tubular creatinine secretion. The serum level of creatinine is influenced by numerous non-renal factors, such as body weight, race, age, and gender. Especially in patients with cirrhosis, the diagnostic value of creatinine may be diminished because of reduced muscle mass.^[24]^ Thus, clinicians should bear in mind that kidney function might already be severely impaired in cirrhosis patients whose serum creatinine surpasses the upper limit of normal and that undetected renal failure may exist even when creatinine remains within reference limits.

Recent studies have uncovered several urinary and plasma biomarkers that can predict kidney injury in patients with cirrhosis.^[14,25,26]^ In particular, increased levels of interleukin-18, kidney injury molecule-1, liver-type fatty acid binding protein, and neutrophil gelatinase-associated lipocalin are specific indicators of structural injury rather than surrogate markers for deceased filtration. Biomarkers reflecting tubular injury have also been associated with such outcomes as worsening of renal impairment and mortality. Recently, Hayek et al.^[27]^ reported that plasma-soluble urokinase-type plasminogen activator receptor was linked to the incidence of chronic kidney disease. We were not able to assess these biomarkers in our cohort due to a lack of urine and serum samples. Further study of additional biomarkers estimating the severity and mortality of renal impairment in cirrhosis is warranted.

We earlier proposed that serum sodium concentration could represent predictive markers of mortality in cirrhosis by Kaplan–Meier statistics.^[28]^ However, sodium concentration did not remain an independent factor of poor prognosis, possibly due to discrepancies in follow-up period and sample number. The limitations of this study are its retrospective nature and small sample size. Moreover, the treatment standards for cirrhosis in Japan changed over the course of follow-up.

## 5 Conclusion

Our findings validated those of global studies on renal impairment as a predictive marker of mortality in patients with cirrhosis who are taking diuretics. Stratification based on the ICA–AKI criteria of kidney function impairment provided similarly good prognostic results, which may help improve the prognosis and disease management of patients with cirrhosis in Japan.

## Acknowledgments

The authors thank Trevor Ralph for his editorial assistance.

## Author contributions

**Conceptualization:** Takeji Umemura.

**Data curation:** Takeji Umemura, Soichiro Shibata, Tomoo Yamazaki, Naoyuki Fujimori.

**Formal analysis:** Takeji Umemura, Akihiro Matsumoto.

**Investigation:** Takeji Umemura, Satoru Joshita, Soichiro Shibata, Tomoo Yamazaki.

**Methodology:** Takeji Umemura, Soichiro Shibata, Akihiro Matsumoto.

**Project administration:** Takeji Umemura, Eiji Tanaka.

**Resources:** Satoru Joshita, Soichiro Shibata, Ayumi Sugiura, Tomoo Yamazaki, Naoyuki Fujimori.

**Software:** Akihiro Matsumoto.

**Supervision:** Eiji Tanaka.

**Validation:** Satoru Joshita, Akihiro Matsumoto.

**Visualization:** Takeji Umemura.

**Writing – original draft:** Takeji Umemura.

**Writing – review & editing:** Satoru Joshita, Eiji Tanaka.

Takeji Umemura orcid: 0000-0001-7985-919X.
